# Epitaxial growth of iridate pyrochlore Nd_2_Ir_2_O_7_ films

**DOI:** 10.1038/srep22282

**Published:** 2016-02-29

**Authors:** J. C. Gallagher, B. D. Esser, R. Morrow, S. R. Dunsiger, R. E. A. Williams, P. M. Woodward, D. W. McComb, F. Y. Yang

**Affiliations:** 1 Department of Physics, The Ohio State University, Columbus, OH 43210, USA; 2 Center for Electron Microscopy and Analysis, Department of Materials Science and Engineering, The Ohio State University, Columbus, OH 43212, USA; 3 Department of Chemistry, The Ohio State University, Columbus, OH 43210, USA; 4 Center for Emergent Materials, The Ohio State University, Columbus, OH 43210, USA

## Abstract

Epitaxial films of the pyrochlore Nd_2_Ir_2_O_7_ have been grown on (111)-oriented yttria-stabilized zirconia (YSZ) substrates by off-axis sputtering followed by post-growth annealing. X-ray diffraction (XRD) results demonstrate phase-pure epitaxial growth of the pyrochlore films on YSZ. Scanning transmission electron microscopy (STEM) investigation of an Nd_2_Ir_2_O_7_ film with a short post-annealing provides insight into the mechanism for crystallization of Nd_2_Ir_2_O_7_ during the post-annealing process. STEM images reveal clear pyrochlore ordering of Nd and Ir in the films. The epitaxial relationship between the YSZ and Nd_2_Ir_2_O_7_ is observed clearly while some interfacial regions show a thin region with polycrystalline Ir nanocrystals.

The 5d transition metal oxides have attracted much attention due to their strong spin-orbit coupling (SOC), which scales with *Z*^4^, where *Z* is the atomic number. Given such strong SOC, exotic phases of matter are predicted to occur, including the topological insulator, Weyl semimetal, and chiral spin liquid^1^,^2^,^3^,^4^. Such materials have potential applications in quantum computing and spintronics^5^. The family of pyrochlore iridate compounds (*A*_2_Ir_2_O_7_) are proving to be a promising arena within which to investigate these interesting phases. In some cases, the materials are geometrically frustrated, as the magnetic species occupy a network of corner sharing tetrahedra. The spin liquid phase has been observed in Pr_2_Ir_2_O_7_ down to 0.3 K^6^, and there is evidence for the Weyl semimetal phase in Rh-doped Nd_2_Ir_2_O_7_^7^ and Eu_2_Ir_2_O_7_^8^. Theoretical studies have shown that the predicted topological insulator and Weyl semimetal phases can be induced by an all-in all-out spin structure of the rare earth (RE) pyrochlore iridates^9^,^10^. In addition, there has been experimental observation of metal-to-insulator transitions in some RE pyrochlore iridates (*A *= Y, Nd, Sm, or Eu) at the same temperature as the magnetic ordering temperature^9^,^11^,^12^,^13^.

Theoretical studies of the pyrochlore iridate thin films predict that the topological insulator and Weyl semimetal phases can be induced in the pyrochlores from epitaxial strain and finite size effects^9^,^14^ providing motivation to synthesize pyrochlore thin films. Previous research reports the synthesis of Bi_2_Ir_2_O_7_^15^ and Eu_2_Ir_2_O_7_^16^ thin films by pulsed laser deposition. In this paper, we report the synthesis of Nd_2_Ir_2_O_7_ using off-axis magnetron sputtering with *ex situ* post-annealing.

## Synthesis

### Sputter Target Synthesis

Nd_2_O_3_ and IrO_2_ powders were thoroughly mixed then ground for at least 2 hours in stoichiometric ratios with an excess of 5 mol% IrO_2_. The samples were pressed into a pellet, and placed in an alumina tube, which was sealed in an evacuated silica tube to prevent the iridium from volatilizing as the IrO_3_ phase^17^ during heating before forming the ternary pyrochlore Nd_2_Ir_2_O_7_ phase. This would cause the powder to be neodymium rich and lead to the formation of Nd_3_IrO_7_ or Nd_6_Ir_2_O_13_ impurity phases (depending on the oxygen level)^18^,^19^. After sealing in a silica tube, samples were heated for 160 hours at a temperature *T *= 950 °C with a ramping rate of 1 °C/min. Afterwards, the samples were taken out of the silica tubes and heated at *T *= 1125 °C in pure O_2_ at atmospheric pressure in a tube furnace for several days to volatilize the excess iridium away, leaving stoichiometric pyrochlore powders with a lattice constant of 10.373 Å (see supplementary information for further analysis of the powder).

### Thin Film Synthesis

**Figure 1:**
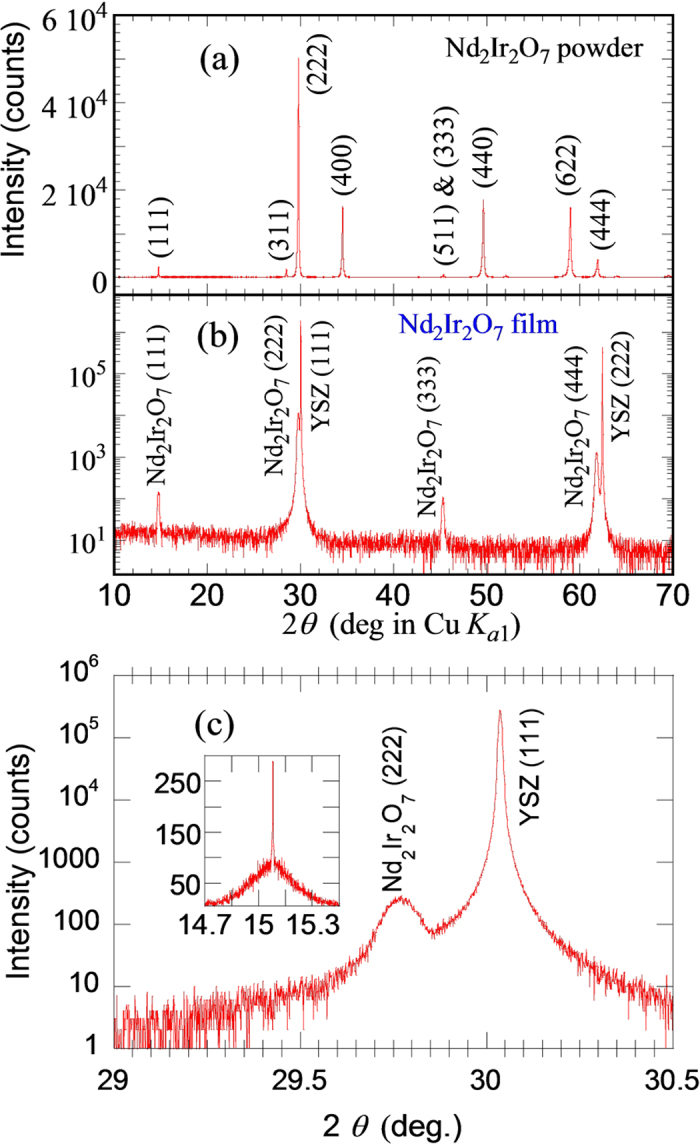
(**a**) *θ*/2*θ* XRD scan of Nd_2_Ir_2_O_7_ powders (taken at Argonne National Lab Advanced Photon Source using an X-ray wavelength of 0.413191 Å), which was converted to the Cu K_*α*1_ wavelength of 1.5405 Å for comparison. (**b**) 2*θ-ω* XRD scan of a 200-nm thick Nd_2_Ir_2_O_7_ film grown on YSZ (111). The sample was post-growth annealed at 750 °C in pure O_2_ at atmospheric pressure with a ramp rate of 0.5 °C/min. (**c**) High resolution XRD scan of the same film around the YSZ (111) and Nd_2_Ir_2_O_7_ (222) peaks. The inset is the rocking curve of the Nd_2_Ir_2_O_7_ (222) peak, which consists of a narrow peak with full-width-at-half-maximum of 0.006° and a broad baseline.

The Nd_2_Ir_2_O_7_ powder was pressed into a 2-inch sputtering target and used for ultra-high vacuum, off-axis magnetron sputtering. Nd_2_Ir_2_O_7_ films were deposited on (111)-oriented 8 mol% yttria-stabilized zirconia (YSZ) substrates in 12.5 mTorr of Ar + 1% O_2_ atmosphere at room temperature. DC sputtering with a constant current of 60 mA was used, resulting in a deposition rate of 2 nm/minute. After the sputter deposition, the films were crystallized by annealing in atmospheric pressure N_2_/O_2_ mixture with oxygen partial pressures ranging from 30 mbar to 200 bar at 750 °C for times ranging from 5 min to 50 hr with ramping rates of 0.5–1 °C/min above 500 °C. Results showed that changing the oxygen partial pressure does not have an obvious effect on the crystal quality; however, annealing at higher temperatures (≥775 °C) results in the formation of the possible Nd_3_IrO_7_ or Nd_6_Ir_2_O_13_ impurity phases. A summary of the effect of annealing under different conditions is shown in Table 1. Each film was analyzed with a Bruker D8 Discover triple-axis X-ray diffractometer. Figure 1(a) shows the synchrotron XRD scan of the Nd_2_Ir_2_O_7_ powder converted to the Cu K_α1_ wavelength for comparison with Fig. 1(b,c) showing the 2*θ-ω* XRD scans of a Nd_2_Ir_2_O_7_ film, which only exhibit the (111)-series of the pyrochlore peaks, indicating a phase-pure, (111)-oriented pyrochlore film on YSZ. The out-of-plane lattice constant was calculated to be 10.387 Å for the Nd_2_Ir_2_O_7_ film, which is close to the bulk value of 10.373 Å obtained from the synchrotron XRD data. The inset to Fig. 1(c) shows a rocking curve of the Nd_2_Ir_2_O_7_ (222) peak which reveals a narrow peak with a full-width-at-half-maximum of 0.006° and a broad baseline possibly caused by inhomogeneity in the Nd_2_Ir_2_O_7_ film.

### Microscopy Analysis and Discussion

**Figure 2:**
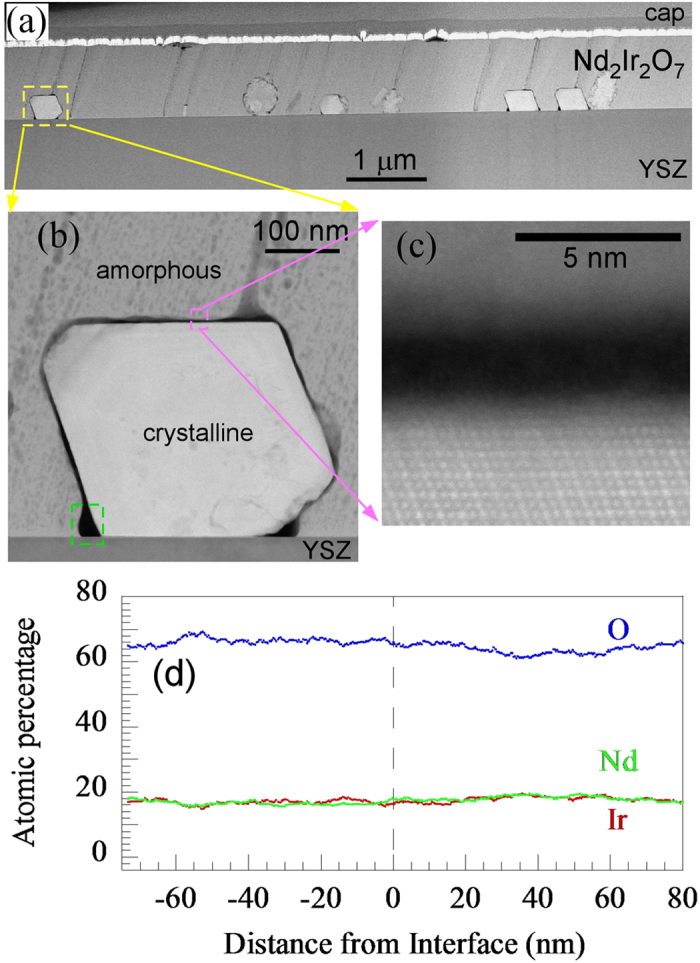
(**a**) STEM image of a Nd_2_Ir_2_O_7_ film with 5 minute post-growth annealing reveals several Nd_2_Ir_2_O_7_ crystallites nucleated at the YSZ interface. (**b**) STEM image of the left-most crystallite in (**a**) with facets along the {111} and {001} planes. (**c**) High magnification STEM image near the top of the crystallite in (**b**). The decrease in volume from the crystallization causes void regions to appear around the crystallite. (**d**) EDX spectra across the amorphous to crystalline transition region with the crystalline region on the right. There is no detected difference in stoichiometry between the regions.

In order to study the early stages of the growth mechanism of Nd_2_Ir_2_O_7_, an 800 nm Nd_2_Ir_2_O_7_ film was deposited, followed by a 5-minute post-growth annealing at 750 °C (with the same ramp rate specified above). High-angle annular dark field scanning transmission electron microscopy (HAADF-STEM) of the pyrochlore film was performed using an FEI probe-corrected Titan^3^ 80–300 S/TEM at 300 kV with a detector collection range of 55–375 mrad. Figure 2(a) shows a low magnification HAADF-STEM image of a cross-section of the annealed film. The film was predominantly amorphous with crystallites nucleated at the YSZ interface. Close examination reveals {111} and {001} faceted crystallites [Fig. 2(b)] surrounded by regions exhibiting dark contrast [Fig. 2(c)] in the HAADF image.

To fully understand the nature of the faceted crystals and Nd_2_Ir_2_O_7_/YSZ interface, elemental analysis was performed by energy-dispersive X-ray (EDX) spectroscopy using the FEI Super-X quad-silicon drift detector system. An EDX profile across the crystalline/amorphous Nd_2_Ir_2_O_7_ interface [Fig. 2(d)] was quantified using experimental Cliff-Lorimer *k*-factors obtained from the Nd_2_Ir_2_O_7_ crystal assuming perfect 2:2:7 stoichiometry. This demonstrates that the as-deposited (amorphous) material was stoichiometric, which implies that the nanocrystals formed during the post-anneal. Note that spectra were collected at specimen orientations far from major crystallographic zone axes to minimize probe-channeling effects that could differ significantly between the amorphous and crystalline materials.

**Figure 3:**
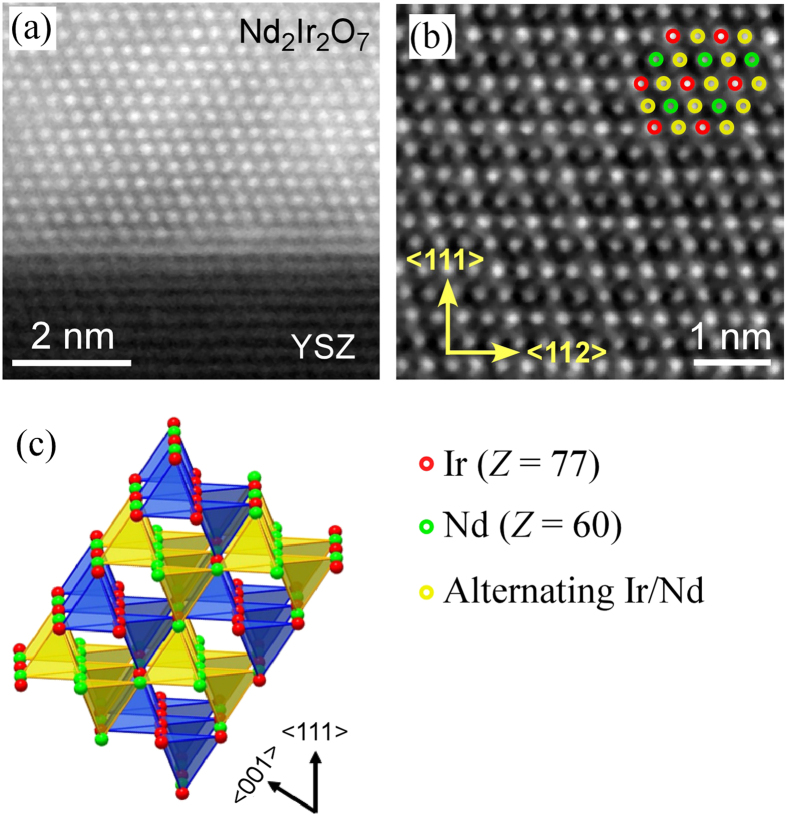
(**a**) STEM image of a Nd_2_Ir_2_O_7_ film grown on YSZ (111) looking down the <110> axis. The Nd_2_Ir_2_O_7_ film is ordered in pyrochlore structure and has an epitaxial relationship with the YSZ substrate. (**b**) High resolution STEM image of the Nd_2_Ir_2_O_7_ film away from the interface shows the pyrochlore ordering of the Nd and Ir sublattices as schematically shown in (**c**).

Films that had been post-annealed for significantly longer times (approximately 12 hours) were observed to be completely crystalline. Higher magnification HAADF-STEM images of the Nd_2_Ir_2_O_7_/YSZ interface in these films [Fig. 3(a)] reveals an epitaxial relationship: <111>_Nd2Ir2O7_ ‖ <111>_YSZ_ and <110>_Nd2Ir2O7_ ‖ <110>_YSZ_. In HAADF-STEM or “Z-contrast” imaging, the intensity of an atomic column is approximately proportional to *Z*^1.8^. As shown in Fig. 3(b,c), this contrast in the images of the Nd_2_Ir_2_O_7_ film is characteristic of a high degree of pyrochlore ordering. The most intense atomic columns contain only Ir atoms (*Z *= 77), while the least intense contain only Nd (*Z *= 60), and the columns of intermediate contrast are alternating Nd and Ir atoms with 1:1 ratio.

**Figure 4:**
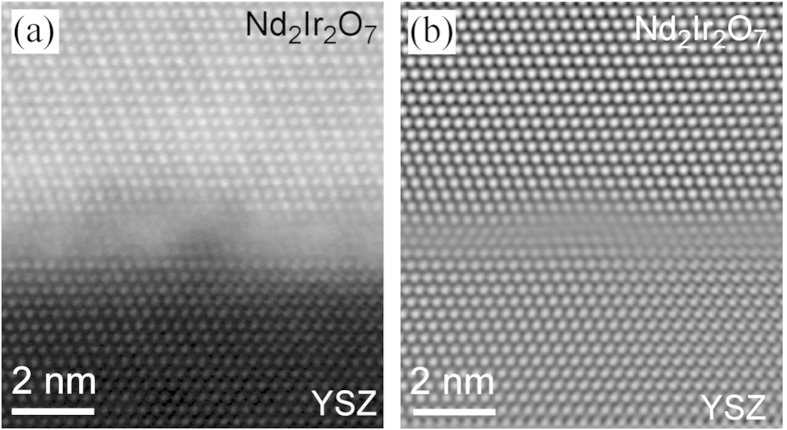
(**a**) STEM image revealing a “cloudy” interface in a region of the sample. (**b**) Fourier filtering of (**a**) reveals an epitaxial relationship at the interface despite the “cloudy” layer.

**Figure 5:**
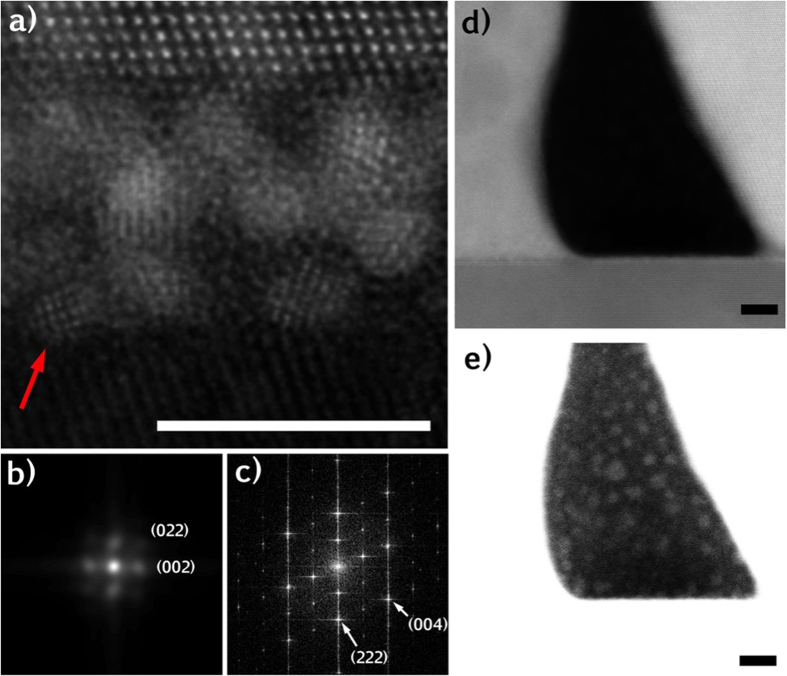
(**a**) High magnification STEM image of nanoparticles at the Nd_2_Ir_2_O_7_/YSZ interface. (**b**) FFT of the nanoparticle indicated by red arrow in (**a**) clearly indicates a cubic structure, with spatial frequencies matching the lattice parameter of metallic Ir, which is distinct from (**c**) the FFT of the pyrochlore Nd_2_Ir_2_O_7_. (d) HAADF STEM image of the bottom-left corner of the island marked by the green box in Fig. 2(b) A saturated image of (**d**) shows metallic Ir nanoparticles filling the void space at the island growth front. Scale bars on all images are 5 nm.

In some regions of the Nd_2_Ir_2_O_7_/YSZ interface, a thin “cloudy” layer was observed in the HAADF-STEM images, as shown in Fig. 4(a). This observation might be mis-interpreted as an amorphous layer at the interface between the YSZ and the Nd_2_Ir_2_O_7_, but this would be inconsistent with the epitaxial relationship described above. In order to demonstrate that this epitaxial relationship remains despite the presence of the “cloudy” layer, the image in Fig. 4(a) was Fourier filtered to enhance the contrast from the crystalline materials. In the Fourier filtered image, the epitaxial relationship is clear [Fig. 4(b)], although the filtering process does reduce the *Z*-contrast and introduces mathematical artifacts around the edges of the image. HAADF-STEM images acquired from thinner TEM foils, Fig. 5(a), revealed nanocrystals, approximately 1-2 nm in diameter in the interfacial region. The nanocrystals exhibit a cubic crystal structure, with spatial frequencies in the fast Fourier Transform (FFT) that match the lattice parameter of metallic Ir [Fig. 5(b)] and are distinctly different from those of the Nd_2_Ir_2_O_7_ film [Fig. 5(c)]. When the dark contrast observed at the triple junction of the YSZ substrate, amorphous Nd_2_Ir_2_O_7_ and crystalline faceted Nd_2_Ir_2_O_7_ noted in Fig. 2(b), is examined at higher magnification [Fig. 5(d)], it is observed that the Ir nanoparticles are also present [Fig. 5(e)].

**Figure 6:**
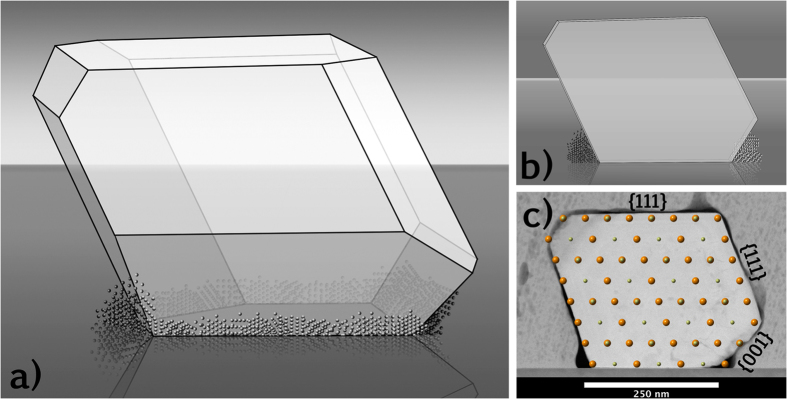
(**a**) Schematic of the crystallite shown in Fig. 2(b) assuming the crystallite forms facets primarily along {111} and {001} planes. Features at the Nd_2_Ir_2_O_7_/YSZ/amorphous triple junction represent Ir nanocrystals. (**b**) Schematic of the sample after being sliced into a <110> TEM foil with Ir nanocrystals visible only at the edges. (**c**) STEM image of crystallite with a schematic of the pyrochlore structure overlaid on the crystallite and the miller indices of the facets labeled. This confirms the crystal facets primarily on {111} planes, but also on {001} planes.

The accumulation of Ir nanocrystals, specifically at the triple junction, can help explain the appearance of Ir metal in some regions of the interface but not others due to projection considerations in STEM imaging. It is reasonable to believe that the dark triple junction seen in Fig. 5(d) completely surrounds the base of the crystalline Nd_2_Ir_2_O_7_ island [Fig. 6(a)]. Consequently, some areas of the TEM specimen near the interface may include partially the dark disordered region and partially crystalline Nd_2_Ir_2_O_7_ along the electron beam path, resulting in a “cloudy” interface, as shown in Fig. 6(a–c).

It is clear that the nucleation and growth of crystalline Nd_2_Ir_2_O_7_ films is influenced by multiple factors. During the annealing process, it is common in some systems for nucleation sites to form at all edges of the film and not exclusively at the interface, resulting in the formation of a polycrystalline thin film, such as the case for YSZ grown on sapphire^20^. In other systems, nucleation sites at the interface grow epitaxially accompanied by the formation of a random polycrystalline film far from the interface, such as the case for YBa_2_Cu_3_O_7_ films grown on SrTiO_3_ with *ex situ* annealing^21^. YSZ is well lattice matched to Nd_2_Ir_2_O_7_ and its cubic fluorite structure is a variant of the pyrochlore structure favoring the formation of nucleation sites exclusively at the interface with an epitaxial orientation. This potentially enables the entire film to be epitaxial as the nucleation sites grow by consuming the surrounding materials until the film is fully crystallized and the epitaxial orientation is well preserved. The curved surfaces that are apparent at the triple junctions [Figs 2(b) and 5(e)] are consistent with wetting phenomena suggesting liquid- or glassy-like behavior at the amorphous-crystalline interface at elevated temperatures. This is worth considering further as the nominal annealing temperature is well below the melting temperature of Nd_2_Ir_2_O_7_. We note that the crystallization of the pyrochlore phase is an exothermic reaction with an enthalpy of formation of 88.7 *kJ/mol*^19^. According to the Neumann–Kopp rule^22^ the heat capacity of Nd_2_Ir_2_O_7_ is


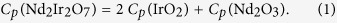


Previous research^23^ has determined the heat capacity of IrO_2_ to be


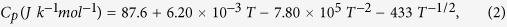


and Nd_2_O_3_ has been determined^24^ to be


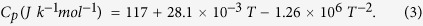


Eqs. (1, 2, 3) predict that the energy released from the crystallization could raise the temperature in the vicinity of the interface from 750 °C to about 1030 °C. This is above the decomposition temperature of IrO_2_ of 1020 °C^19^, thus causing the formation of metallic Ir nanoparticles near the crystalline/amorphous interface. The high oxygen ion conductivity of YSZ, which peaks at 8 mol% Y_2_O_3_^25^, may enhance the formation of Ir nanoparticles by creating an oxygen deficient interfacial layer. It may be possible to prevent such an interfacial layer by using a buffer material to block the oxygen flow to the substrate. In addition, it may be possible to prevent IrO_2_ decomposition by annealing at lower temperatures; however, crystallizing Nd_2_Ir_2_O_7_ at temperatures below 750 °C is challenging.

## Conclusions

In summary, Nd_2_Ir_2_O_7_ powders were synthesized in an evacuated silica tube and their structural properties were analyzed using laboratory as well as synchrotron X-ray diffraction. We report growth of Nd_2_Ir_2_O_7_ epitaxial films using off-axis magnetron sputtering followed by post-annealing. XRD results demonstrate phase-pure epitaxial films grown on YSZ (111). HAADF-STEM images reveal clear pyrochlore ordering. Additionally, Nd_2_Ir_2_O_7_ grows by the formation of epitaxial nucleation sites exclusively at the YSZ interface that slowly absorb the surrounding amorphous material forming a (111)-oriented crystalline film.

## Additional Information

**How to cite this article**: Gallagher, J. C. *et al*. Epitaxial growth of iridate pyrochlore Nd_2_Ir_2_O_7_ films. *Sci. Rep.* **6**, 22282; doi: 10.1038/srep22282 (2016).

## Supplementary Material

Supplementary Information

## Figures and Tables

**Table 1:** Summary of results of annealing amorphous Nd_2_Ir_2_O_7_ films.

O_2_ partial pressure effect (12 hour anneal at *T *= 750 °C)	
0.03 bar to 200 bar O_2_	Pure phase epitaxial pyrochlore Nd_2_Ir_2_O_7_ films
No O_2_ (Pure N_2_ or vacuum)	No Nd_2_Ir_2_O_7_ phase detected
Temperature effects (12 hour anneal at 1 bar of pure O_2_)	
*T *≥ 775 °C	Epitaxial Nd_2_Ir_2_O_7_ forms, but with an impurity phase at 2*θ* 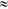 29° in XRD (possibly Nd_3_IrO_7_ or Nd_6_Ir_2_O_13_)
*T *= 750 °C	Pure phase epitaxial pyrochlore Nd_2_Ir_2_O_7_ films
*T *= 700 °C	Pure phase epitaxial pyrochlore Nd_2_Ir_2_O_7_ films, though intensities of the pyrochlore XRD peaks are weaker and can be enhanced by further heating at 750 °C
Annealing Time Effects (750 °C in air)	
5 minutes	Nucleation of Epitaxial Nd_2_Ir_2_O_7_ crystallites of ~100 nm observed at YSZ interface [see Fig. 2(a)].
≥6 hours	Nd_2_Ir_2_O_7_ films are fully crystalized
