# Supplementary material for: Epitaxial growth of iridate pyrochlore Nd2Ir2O7 films
Source: Sci Rep. 2016 Feb 29;6:22282. doi: 10.1038/srep22282 (PMC4770277; doi:10.1038/srep22282)
Supplement: Supplementary Information [file srep22282-s1.pdf]

Supplementary Material for "Epitaxial growth of iridate pyrochlore $Nd_2Ir_2O_7$ films"

J. C. Gallagher[1], B. D. Esser[2], R. Morrow[3], S. R. Dunsiger[1], R. E. A. Williams[2], P. M. Woodward[3], D. W. McComb[2], and F. Y. Yang[1]

[1]Department of Physics, The Ohio State University, Columbus, OH, 43210, USA

[2]Center for Electron Microscopy and Analysis, Department of Materials Science and Engineering, The Ohio State University, Columbus, OH, 43212, USA

[3]Department of Chemistry, The Ohio State University, Columbus, OH, 43210, USA

Supplementary Material

The powders were analyzed on beamline 11-BM at the Advanced Photon Source (APS), Argonne National Laboratory using high-resolution synchrotron powder x-ray diffraction (XRD) using a wavelength of 0.413191 Å with a step size of 0.001° and a scan speed of 0.01°/s, as shown in Fig. S1 for $Nd_2Ir_2O_7$. Rietveld refinement analysis was conducted utilizing the GSAS-II software package.[S1] The inset to Fig. S1 shows that the diffraction peaks are asymmetrical. The asymmetry had significant 2θ dependence, revealing a continuous range of lattice constants in the sample possibly due to an oxygen off-stoichiometry distribution near the surface of the particles. The asymmetry could not be accounted for with realistic instrumental parameters. We modeled the asymmetric peak shape with a three-phase fit utilizing different lattice parameters: one primary phase with a refined smaller lattice constant and two oxygen-deficient pyrochlore phases with slightly larger fixed lattice constants. The remaining refinement parameters were constrained to be

equivalent in each phase. The results of the fit are shown in Figs. S1. The refined lattice parameters and locations of the atoms in $Nd_2Ir_2O_7$ powders are reported in Table S1.

Magnetization (χ) measurements on the powder were taken using a Quantum Design superconducting quantum interface device (SQUID) magnetometer at a magnetic field H = 100 Oe and at temperatures ranging from 2 to 300 K with both field and zero field cooling protocols [Fig. S2 (a)]. The temperature dependence appears roughly Curie like (1/T) in character. However, by taking the difference between the field and zero field cooled measurements [Fig. S2 (b)], two features likely associated with magnetic phase transitions emerge: a stronger one at 34 K and a more subtle one at 119 K. The nature of the magnetic ordering transition is a topic of continuing debate. For simpler pyrochlores with only one magnetic species such as in $Eu_2Ir_2O_7$ and $Y_2Ir_2O_7$, clear magnetic ordering of the Ir site to an all-in-all-out structure has been reported around 120 K.[S2–S6] $Nd_2Ir_2O_7$ is more complicated due to the Nd on the A site also having a magnetic moment. Neutron diffraction is sensitive to the larger Nd moment and reveals Nd ordering around 15 K.[S7, S8] However this technique is much less sensitive to the much smaller iridium moment and did not observe any evidence of higher temperature ordering.[S9] It is known other (Mo based) pyrochlores with multiple magnetic species may have multiple ordering transitions temperatures.[S10] Since the Ir-Ir exchange interactions are stronger than those between Nd-Nd or Nd-Ir due to the greater spatial extent of 5d transition metal electronic orbitals, it is anticipated that the Ir sublattice would undergo magnetic ordering at a higher temperature. Therefore, we speculate that the ~120 K transition may be due to an onset of Ir ordering while the 34 K transition represents the onset of Nd spin freezing. The roughly Curie-like temperature dependence of the magnetization is thus interpreted as arising from paramagnetic contributions of the larger Nd moment which persist below the Ir ordering transition. At lower temperatures the interplay between the two sublattices

becomes more significant. Though our 34 K transition temperature agrees with previous research,[S11] there is considerable variation in the temperature at which this Nd ordering occurs.[S5, S7, S8] We hypothesize that this is due to the effect of the ionic radius of the rare earth ion on magnetic ordering temperature. The magnetic ordering temperature in the pyrochlore iridates depends heavily on the *A*-site ionic radius. The ionic radius of the Nd is such that the system is close to the transition to a spin liquid phase which does not order at low temperatures.[S12] Therefore, small variations in lattice constant due to sample stoichiometry or lattice strain may cause significant change in magnetic ordering temperature.

References

S1. Toby, B. H. & Von Dreele, R. B. GSAS-II : the genesis of a modern open-source all purpose crystallography software package. *J. Appl. Crystallogr.* **46,** 544–549 (2013).

S2. Taira, N., Wakeshima, M. & Hinatsu, Y. Magnetic properties of iridium pyrochlores $R_2Ir_2O_7$ (R = Y , Sm , Eu and Lu). *J. PhysicsCondensed Matter* **5527,** 4427–5533 (2001).

S3. Liu, X. *et al.* Perpendicular magnetization reversal, magnetic anisotropy, multistep spin switching, and domain nucleation and expansion in Ga 1-xMn xAs films. *J. Appl. Phys.* **98,** 0–11 (2005).

S4. Sagayama, H. *et al.* Determination of long-range all-in-all-out ordering of Ir4+ moments in a pyrochlore iridate $Eu_2Ir_2O_7$ by resonant x-ray diffraction. *Phys. Rev. B - Condens. Matter Mater. Phys.* **87,** 100403 (2013).

S5. Disseler, S. M. *et al.* Magnetic order and the electronic ground state in the pyrochlore iridate $Nd_2Ir_2O_7$. *Phys. Rev. B* **85,** 174441 (2012).

S6. Zhao, S. *et al.* Magnetic transition, long-range order, and moment fluctuations in the pyrochlore iridate $Eu_2Ir_2O_7$. *Phys. Rev. B - Condens. Matter Mater. Phys.* **83,** 2–5 (2011).

S7. Ueda, K. *et al.* Magnetic field-induced insulator-semimetal transition in a pyrochlore $Nd_2Ir_2O_7$. *Phys. Rev. Lett.* **115,** 056402 (2015).

S8. Tomiyasu, K. *et al.* Emergence of magnetic long-range order in frustrated pyrochlore

$Nd_2Ir_2O_7$ with metal-insulator transition. *J. Phys. Soc. Japan* **81,** 034709 (2012).

S9. Shapiro, M. C. *et al.* Structure and magnetic properties of the pyrochlore iridate $Y_2Ir_2O_7$. *Phys. Rev. B* 1–5 (2012). doi:10.1143/JPSJ.71.2578

S10. Ali, N., Hill, M. ., Labroo, S. & Greedan, J. . Magnetic and electrical properties of $R_2Mo_2O_7$ pyrochlore compounds. *J. Solid State Chem.* **83,** 178–187 (1989).

S11. Ueda, K. *et al.* Variation of Charge Dynamics in the Course of Metal-Insulator Transition for Pyrochlore-Type $Nd_2Ir_2O_7$. *Phys. Rev. Lett.* **109,** 136402 (2012).

S12. Matsuhira, K., Wakeshima, M., Hinatsu, Y. & Takagi, S. Metal–Insulator Transitions in Pyrochlore Oxides $Ln_2Ir_2O_7$. *J. Phys. Soc. Japan* **80,** 094701 (2011).

Table S1: Rietveld refinement results of the synchrotron XRD data for $Nd_2Ir_2O_7$ powders, which give the *xyz* coordinates of each atom in the unit of lattice constant *a*. The data was refined with the 3 phases of $Nd_2Ir_2O_7$ with different lattice constants (dominate phase in bold), but with atomic parameters constrained to be equivalent. The two less prevalent phases had larger fixed lattice parameters while the dominant phase *a* was refined. The three lattices constants used in each phase are displayed, with the dominate phase listed first. The fitting quality is described by R_{wp} = 12.83 %, R_p = 9.09 %, and χ^2 = 4.338.

	x	*y*	*z*	Site	U_{iso} (Å^2)	Lattice constant *a* (Å)
Nd	0	0	0	16c	0.00779(7)	**10.37323(3)**<br>10.400<br>10.430
Ir	0.5	0.5	0.5	16d	0.00303(4)	
O	0.125	0.125	0.125	8a	0.007(1)	
O	0.4199(3)	0.125	0.125	48f	0.007(2)	

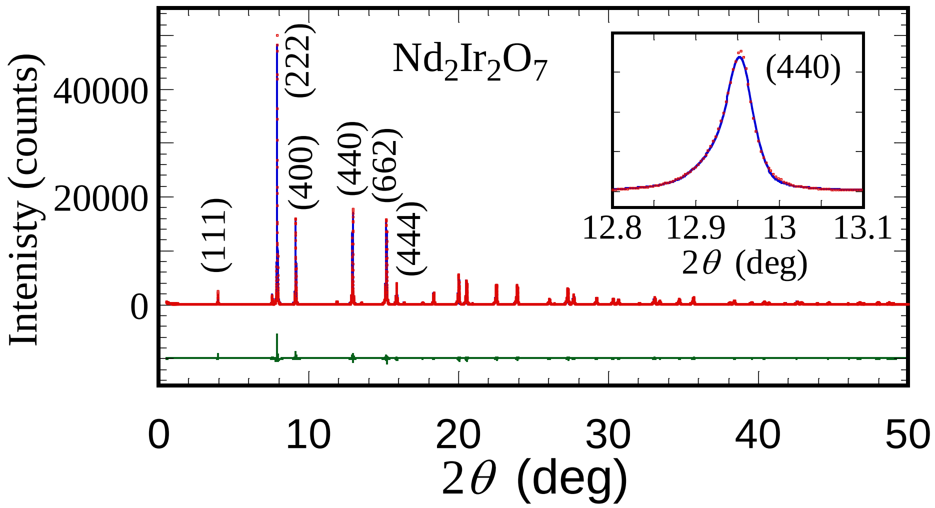

Figure S1. θ/2θ XRD scans (red symbols) of $Nd_2Ir_2O_7$ powders taken at the Argonne National Laboratory's Beam Line 11 with x-ray wavelength of 0.413191 Å. The Rietveld refinements (blue curve) give the structural parameters shown in Table I. The green curve is the difference between experimental data (red) and calculated pattern (blue). The insets show the pyrochlore (440) peaks where the asymmetry is due to the inhomogeneous oxygen stoichiometry in the powders.

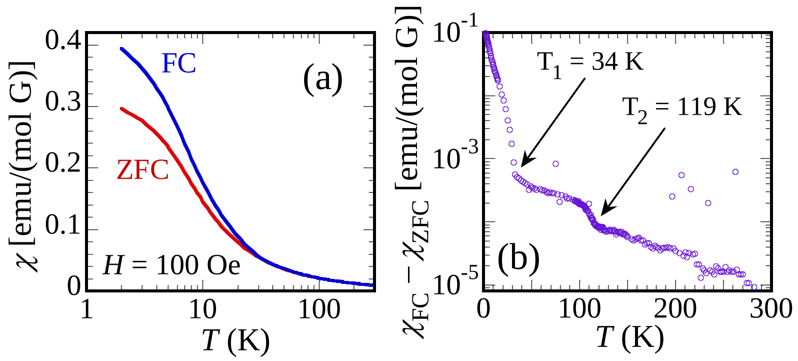

Figure S2: (a) Magnetic susceptibility of the $Nd_2Ir_2O_7$ powders measured at H = 100 Oe for both FC and ZFC samples. (b) The difference between ZFC and FC curves in (b) indicated two magnetic phase transitions, the first at 34 K and the second at 119 K.
